# Molecular Insight into the Recognition of DNA by the DndCDE Complex in DNA Phosphorothioation

**DOI:** 10.3390/ijms26125765

**Published:** 2025-06-16

**Authors:** Wencheng Fu, Yuli Wang, Yashi Ge, Haiyan Gao, Xuan Sun, Zixin Deng, Lianrong Wang, Shi Chen, Xinyi He, Geng Wu

**Affiliations:** 1 State Key Laboratory of Microbial Metabolism, School of Life Sciences & Biotechnology, the Joint International Research Laboratory of Metabolic & Developmental Sciences MOE, Shanghai Jiao Tong University, Shanghai 200240, China; fwc5498085@sjtu.edu.cn (W.F.); andrea_wang521@sjtu.edu.cn (Y.W.); sunshine.1997@sjtu.edu.cn (X.S.); zxdeng@sjtu.edu.cn (Z.D.); xyhe@sjtu.edu.cn (X.H.); 2 Key Laboratory of Combinatorial Biosynthesis and Drug Discovery, Ministry of Education, School of Pharmaceutical Sciences, Wuhan University, Wuhan 430071, China; 2021203060030@whu.edu.cn (Y.G.); ghy@whu.edu.cn (H.G.); lianrong@whu.edu.cn (L.W.); shichen@whu.edu.cn (S.C.)

**Keywords:** DndC, DndCDE complex, DNA-DndCDE complex, specificity loop, phosphorothioation modification

## Abstract

In a vast variety of prokaryotes such as *Escherichia coli* and *Streptomyces lividans*, the DNA degradation (Dnd) CDE protein complex (consisting of DndC, DndD, and DndE), together with the DndA/IscS protein and the DndFGH complex, function as a defense barrier to prevent the invasion of non-self-DNA. The DndCDE complex introduces phosphorothioation (PT) modifications into DNA, and the DndFGH complex specifically cleaves non-PT DNA and, thus, restricts horizontal gene transfer and phage invasion. Despite the central importance of the DndCDE complex in DNA PT modification, which catalyzes the oxygen–sulfur swap on DNA, our understanding of this key complex remains poor. Here, we employed protein structure prediction to provide a reasonably reliable prediction of the structure of the DndCDE complex and a 23 bp DNA-DndCDE complex. We found that among the three proteins in the DndCDE complex, DndC, especially its “specificity loop”, plays a key role in recognizing the consensus PT modification sequence. In addition, the DndD protein is found to possess a highly conserved structural surface on its globular domain, presumably mediating the dimerization of DndD as well as the DndCDE complex. Furthermore, our normal mode analysis showed that there exists a dynamic transition between a closed and an open state for the DndCDE complex, facilitating its association and release of DNA. Our conclusions were corroborated by biochemical assays using purified proteins. On the whole, we provide molecular insights into the assembly and DNA-recognition mechanism of a central protein complex involved in DNA phosphorothioation.

## 1. Introduction

Epigenetic modifications on DNA, such as methylation and hydroxymethylation, have been found in all organisms [1]. DNA phosphorothioation (PT) is a unique kind of DNA epigenetic modification that occurs on the sugar–phosphate backbone rather than the bases of DNA, and it has been found to be widespread among prokaryotes, including both bacteria and archaea [2,3,4,5,6]. During DNA PT modification, a non-bridging oxygen atom in the *R*_p_ configuration on the phosphodiester backbone of DNA is specifically replaced by sulfur [7]. Four consensus sequence patterns of DNA PT modifications have been found: G_PT_AAC/G_PT_TTC in *Escherichia coli* B7A, *Salmonella enterica* Cerro 87, and *Vibrio tasmaniensis* 1F-267; G_PT_GCC in *Streptomyces lividans* 1326, *Vibrio splendidus* ZS-139, *Pseudomonas fluorescens* Pf0-1, and *Geobacter uraniumreducens* Rf4; G_PT_ATC in *Hahella chejuensis* KCTC2396 and *Bermanella marisrubri* RED65; and C_PT_CA in *Vibrio cyclitrophicus* FF75 [8,9,10].

The DndC-DndD-DndE (referred to as DndCDE hereafter) protein complex, which is encoded by the *dnd* gene cluster in the bacterial genome, is responsible for introducing the sulfur atom into DNA in three kinds of double-strand DNA PT modifications: G_PT_AAC/G_PT_TTC, G_PT_GCC/G_PT_GCC, and G_PT_ATC/G_PT_ATC. It catalyzes the oxygen–sulfur swap on DNA and, therefore, plays a central role in DNA PT modification [11,12]. Crystal structures of DndE from bacteria and archaea have been determined, which show that DndE contains positively charged surfaces, which might participate in DNA binding and DNA nicking [13,14,15]. DndC has been found to harbor a 4Fe-4S iron–sulfur cluster [16], which accepts the activated sulfur atom from a cysteine desulfurase DndA/IscS [17,18,19,20,21,22]. The DndFGH (DndF-DndG-DndH) protein complex, which is encoded by the *dndFGH* gene cluster next to the *dndCDE* gene cluster, specifically introduces nicking breaks on non-PT DNA instead of PT-DNA and, therefore, restricts the invasion of foreign DNA and protects the host bacterial DNA from damage [23,24]. In *Vibrio cyclitrophicus* FF75 and some other bacteria, the SspABCD system is responsible for the C_PT_CA type of single-strand DNA PT modification [25,26].

Despite the key role of the DndCDE complex in DNA PT modification, our knowledge of this crucial complex remains poor. The structures of DndC, DndD, and the DndCDE complex are still unavailable, and we do not understand how the DndCDE complex recognizes DNA in a consensus sequence-specific pattern. In recent years, there has been a significant breakthrough in protein structure prediction, with AlphaFold2 and AlphaFold3 being able to provide rather accurate predicted structures for both single proteins and protein–protein complexes [27,28]. For example, Coskuner–Weber used AlphaFold3 to model α-synuclein, a key protein involved in neurodegenerative diseases. The results generated conformational ensembles consistent with experimental NMR data, revealing the dynamic nature of intrinsically disordered proteins (IDPs) [29]. In research on the SARS-CoV-2 main protease (Mpro), Aniana et al. utilized AlphaFold3 to predict protein–protein interaction interfaces and investigated alternative dimerization and higher-order assemblies [30]. Chai-1 was specifically employed to predict the three-dimensional structure of the protein–ligand complex. Numerous successful applications have emerged in recent years. For example, Cheng et al. utilized the AI-based protein–ligand prediction tool Chai-1 to construct a structural model of the heteropentameric α1β1 nAChR, providing deep genetic and structural insights into the mechanisms of resistance to neonicotinoid drugs [31]. Huang et al., leveraging Chai-1, successfully identified a covalent bond formed between compound CMNPD31124 and the CYS-190 residue in the protein kinase PKMYT1. This interaction was further supported by additional contacts involving TYR-121 and GLY-122, and subsequent experimental studies validated this prediction, fully demonstrating the practical utility of Chai-1 in structural analysis [32]. Additionally, Medvedev et al. employed various AI-driven protein structure prediction methods—including AlphaFold3 and Chai-1—to successfully elucidate the impact of post-translational modifications (PTMs) on small-molecule binding, highlighting the potential of AI in deciphering complex biological regulatory mechanisms [33]. In this work, we employed a variety of structure prediction methods such as AlphaFold3 and Chai-1 to predict the structures of DndC, DndD, the DndCDE complex, as well as the DNA-DndCDE complex. Interestingly, our results suggest that DndD adopts different conformations, which underlies the ability of the DndCDE complex to cycle between association with DNA and subsequent dissociation. In addition, of the three proteins in the DndCDE complex, DndC was predicted to provide the major and direct contact with DNA, with a loop (named “specificity loop”) inserting into the major groove of consensus DNA modification sequence and playing a key role in DNA phosphorothioation. Finally, our in vitro biochemical assays using purified proteins and in vivo microbiological assays provide reliable verifications for the above findings.

## 2. Results

### 2.1. Structural Prediction of DndC

**Figure 1:**
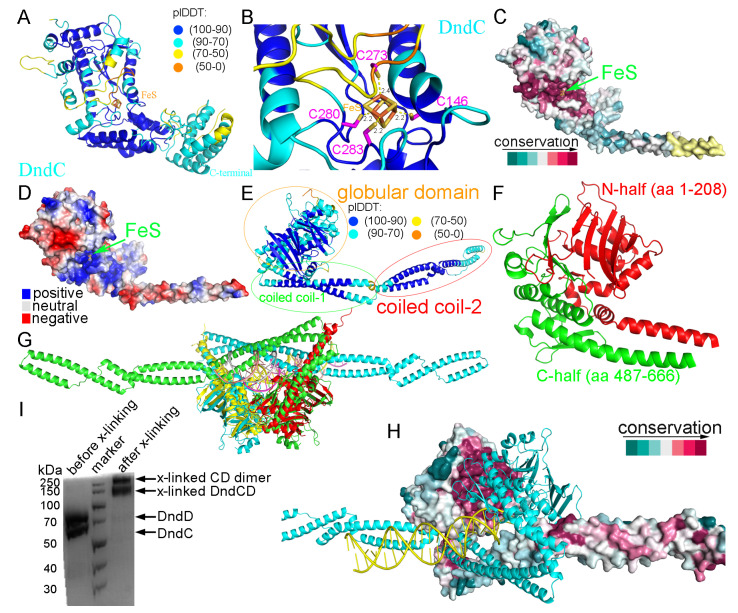
Predicted structures of DndC and DndD. (**A**) Predicted structure of *E. coli* B7A DndC. (**B**) The iron–sulfur cluster and the four conserved cysteine residues coordinating the iron–sulfur cluster. (**C**) The conservation of DndC across different species of prokaryotes was mapped onto the surface of its structure. The green arrow indicates the iron–sulfur cluster. (**D**) The electrostatic surface potential of DndC. (**E**) Predicted structure of *E. coli* B7A DndD. (**F**) Structure of the globular domain of DndD. (**G**) The AlphaFold3-predicted structure of the DndD dimer was structurally aligned with the Rad50 (red and yellow)-DNA (yellow and magenta) crystal structure using molecular superimposition. (**H**) The dimerization interface of DndD is highly conserved. (**I**) Cross-linking experiments using purified proteins of the DndC-DndD complex.

We first employed Chai-1 to predict the structure of DndC from *E. coli* B7A, which consists of 540 amino acid residues with a 4Fe-4S iron–sulfur cluster. The confidence in the structure prediction, as reflected by the ipTM (0.22) and pTM (0.88) scores, was rather high (Figure 1A and Supplementary Table S1). A comparative structural analysis of Chai-1 (cyan, pTM = 0.88) and AlphaFold3 (magenta, pTM = 0.86) predictions for DndC revealed minimal divergence in the core region, with an aligned root mean square deviation (RMSD) of 0.781 Å (Supplementary Figure S1A). While Pu et al. (2020) identified three conserved cysteine residues (Cys146, Cys280, and Cys283) shared between DndC and adenosine 5′-phosphosulfate reductase (APS reductase) for 4Fe-4S cluster coordination, they noted that Cys262 and Cys273—though conserved in DndC—were absent in APS reductase, leaving the fourth coordinating cysteine unresolved [34]. Our Chai-1 prediction resolved this ambiguity by identifying Cys273 as the fourth ligand within a loop region (Figure 1B). Structural alignment of DndC with APS reductase (PDB: 2GOY; RMSD = 3.926 Å) further localized the 4Fe-4S cluster to a spatially analogous region (Supplementary Figure S1B). This establishes that DndC employs four cysteine residues (Cys146, Cys273, Cys280, and Cys283) to coordinate the 4Fe-4S cluster (Figure 1B). Notably, Cys273 resides within a dynamic loop structure exhibiting potential conformational flexibility, which may regulate DNA binding and sulfur modification (Supplementary Figure S1C). This is mechanistically consistent with the role of the fourth iron atom as an active site for accepting activated sulfur from the DndA/IscS protein, requiring direct participation of Cys273 in sulfur transfer. The 4Fe-4S cluster is located in a highly conserved (Figure 1C) and positively charged groove (Figure 1D) at the surface of the DndC structure.

### 2.2. Structural Prediction of DndD

The structure of DndD from *E. coli* B7A (666 amino acid residues) was predicted by AlphaFold3, with a confidence level pTM score of 0.67 (Figure 1E and Supplementary Table S1). It consists of three domains: a globular domain (residues 1–208 and 487–666), a coiled coil domain (named as coiled coil-1, residues 209–263 and 426–486), and a second coiled coil domain (named as coiled coil-2, residues 264–425). The shape of the globular domain of DndD is like a sphere comprising two halves, with one half-sphere provided by the N-terminal residues 1–208 and the other half-sphere from the C-terminal residues 487–666 of DndD (Figure 1F). From the AlphaFold3 results, we found that DndD is able to dimerize through its globular domain (Figure 1G). Structural alignment further demonstrated compatibility between the Rad50-DNA complex and the DndD globular domain dimer-DNA assembly (RMSD = 2.820 Å, Figure 1G and Supplementary Table S2). A structural analysis of the *Methanococcus jannaschii* Mre11/Rad50 (MR) complex reveals that ATPγS-bound Rad50 nucleotide-binding domains symmetrically accommodate partially deformed DNA within their central groove [35]. Notably, molecular modeling demonstrates striking structural congruence between the Chai-1-predicted ATPγS-bound DndD globular domain dimer-DNA complex and the observed Rad50-DNA-ATPγS architecture (Supplementary Table S1 and Supplementary Figure S2A–D), supporting the evolutionary conservation of this ATP-driven DNA processing mechanism and the reliability of predictions. Evolutionary conservation analysis using the Consurf server revealed pronounced conservation of the dimerization interface (Figure 1H). These findings were corroborated by solution-phase cross-linking experiments using purified DndC-DndD protein complexes (Figure 1I).

### 2.3. The Recognition Mechanism Between DndC and DndD

**Figure 2:**
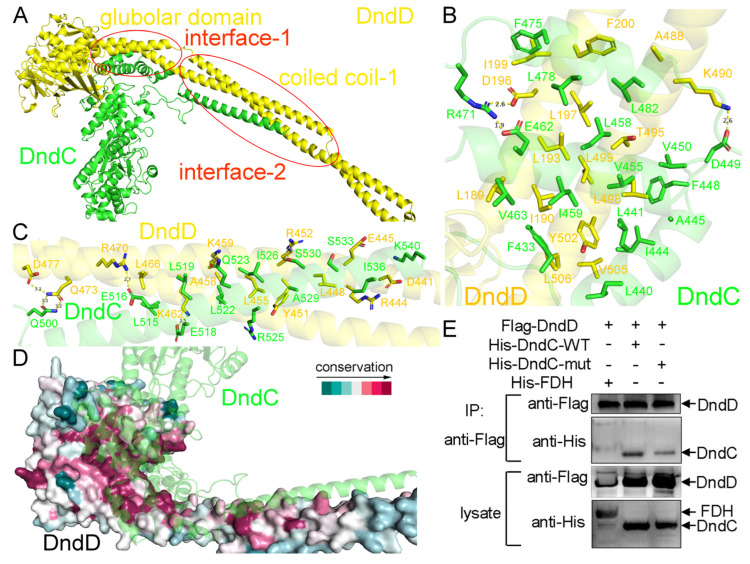
Predicted structure of the DndC-DndD complex. (**A**) Predicted structure of the DndC-DndD complex. There are two interaction interfaces between DndC and DndD, which are highlighted by red boxes in the figure. (**B**) In the first interface, the globular domain of DndD recognizes residues 433–482 of DndC. (**C**) In the second interface, the coiled coil-1 domain of DndD interacts with the very C-terminal α-helix of DndC. (**D**) Both of these two recognition surfaces are highly conserved across various species. (**E**) The nickel column pull-down assay using purified proteins of DndC and DndD.

To understand the recognition mechanism between DndC and DndD, we predicted the structure of the *E. coli* B7A DndC-DndD complex by AlphaFold3, with a quality score of 0.67 (ipTM) + 0.69 (pTM) = 1.36 (Figure 2A and Supplementary Table S1). Two binding interfaces were found between DndC and DndD (Figure 2A). In the first DndC-DndD binding interface, the globular domain of DndD, including residues from both its N- and C-terminal half-spheres, recognizes residues 433–482 of DndC, mainly through hydrophobic interactions among non-polar residues of either protein (Figure 2B). In the second DndC-DndD binding interface, the coiled coil-1 domain of DndD interacts with the very C-terminal α-helix of DndC, using both hydrophobic and hydrogen bonding interactions (Figure 2C). Both of these two recognition surfaces are highly conserved across different species of bacteria and archaea (Figure 2D), suggesting that DndC and DndD in different prokaryotic organisms employ similar interfaces to recognize each other. The organization of the DndC-DndD complex was supported by our electron microscopy results (Supplementary Figure S3).

Using the nickel column pull down assay using purified proteins, we confirmed that DndC binds to DndD constructs with coiled coil-2 deleted (Supplementary Figure S4A) or with both coiled coil-1 and coiled coil-2 deleted (Supplementary Figure S4B). Furthermore, point mutations in DndC (L458D/E462A/R471A) disrupted the interaction between DndC and DndD (Figure 2E). In our electron microscopy results, putative dimers of the DndCDE complex were observed, which was consistent with our conclusion that DndD is able to dimerize (Supplementary Figure S5).

### 2.4. The Recognition Mechanism Between DndD and DndE

**Figure 3:**
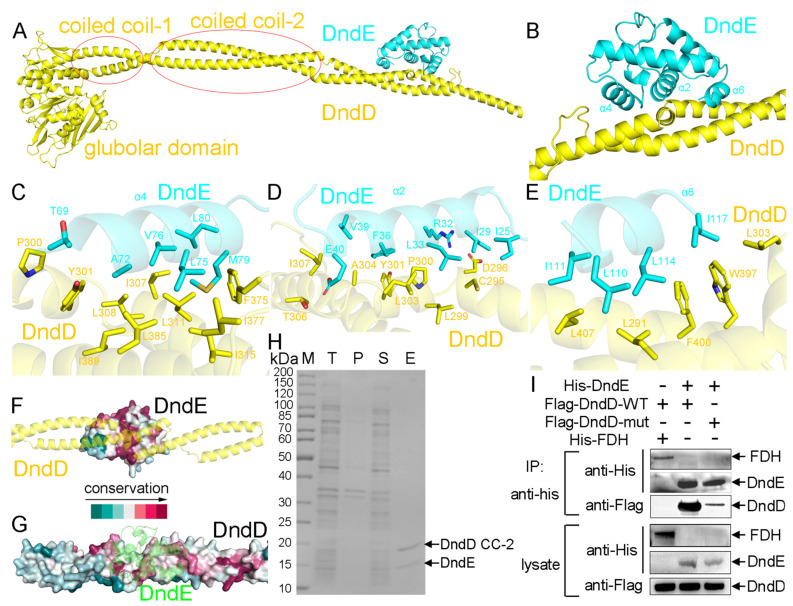
DndD employs its coiled coil-2 domain to recognize DndE. (**A**) Predicted structure of the DndD-DndE complex. (**B**) DndE employs its α2, α4, and α6 helices to interact with DndD. (**C**) The binding interface between DndE-α4 and DndD. (**D**) The binding interface between DndE-α2 and DndD. (**E**) The binding interface between DndE-α6 and DndD. (**F**) The DndD-binding surface on DndE is highly conserved. (**G**) The DndE-binding surface on DndD is conserved across various species. (**H**) A pull-down experiment confirmed that the coiled coil-2 domain of DndD was sufficient for interacting with DndE. (**I**) Point mutations on the DndE-binding residues of DndD abolished its interaction with DndE.

Although the crystal structure of DndE was already determined, it is still unclear how DndE and DndD interact with each other. To answer this question, we obtained the predicted structure of the DndD-DndE complex using AlphaFold3, with a quality score of 0.88 (ipTM) + 0.37 (pTM) = 1.25 (Figure 3A and Supplementary Table S1). In the DndD-DndE complex, only the coiled coil-2 domain of DndD is involved in recognizing DndE, and neither the globular domain nor the coiled coil-1 domain of DndD provides any contact with DndE. Being a small all-α-helical protein, DndE employs three of its α helices, α2, α4, and α6, to interact with DndD (Figure 3B). The binding between DndD and DndE is mainly mediated by hydrophobic interaction, involving non-polar residues from both proteins (Figure 3C–E). Both the DndD-binding surface on DndE (Figure 3F) and the DndE-binding surface on DndD (Figure 3G) are conserved across various species. In addition, our pull-down experiment confirmed that the coiled coil-2 domain of DndD is sufficient for interacting with DndE (Figure 3H), and point mutations on the DndE-binding residues of DndD abolished its interaction with DndE (Figure 3I).

### 2.5. Recognition of DNA by the DndCDE Complex

**Figure 4:**
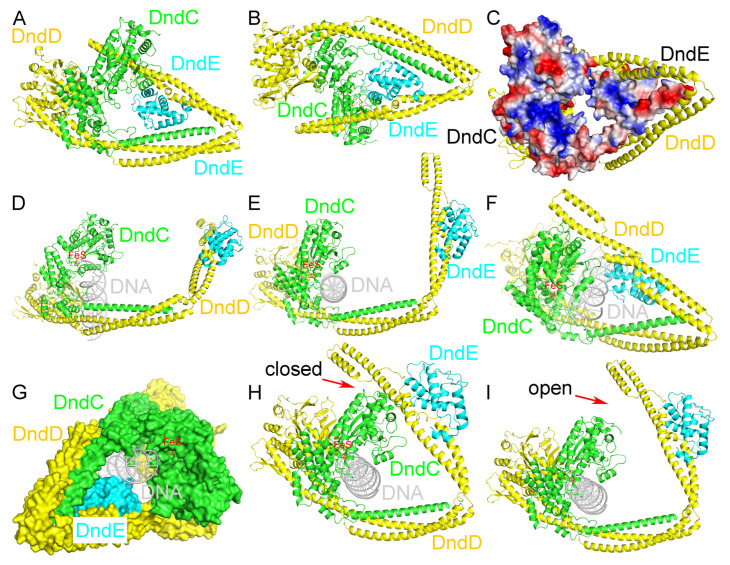
Recognition of DNA by the DndCDE complex. (**A**) Predicted structure of the DndCDE complex. (**B**) Predicted structure of the DndCDE complex viewed from a different angle. (**C**) DndC and DndE provide the inner positively charged surface of the ring-like structure of the DndCDE complex, and DndD is located at the outside of the ring. (**D**) Side view of the predicted structure of *E. coli* B7A DndCDE in a complex with 23 bp of DNA (Supplementary Tables S1 and S2). (**E**) Top view of the predicted DndCDE-DNA complex structure (Supplementary Tables S1 and S2). (**F**) Oblique view of the predicted DndCDE-DNA complex structure (Supplementary Tables S1 and S2). (**G**) The cylindrical shape of DNA is complementary to that of the hollow cavity of the predicted DndCDE complex, with DndC and DndE contacting DNA (Supplementary Tables S1 and S2). A normal mode analysis confirmed that conformation transition enables the predicted DndCDE complex to cycle between association (**H**) with and dissociation (**I**) from DNA. The red arrows indicate the closed state (**H**) where the complex is associated with DNA, and the open state (**I**) where it is dissociated from DNA.

We further obtained the predicted structure of the DndCDE complex using AlphaFold3, with a quality score of 0.52 (ipTM) + 0.61 (pTM) = 1.13 (Figure 4A,B and Supplementary Table S1). The DndCDE complex forms a ring-like structure, with DndD binding to both DndC and DndE. DndC and DndE provide the inner surface of the ring-like structure of the DndCDE complex, which is positively charged, and DndD is located at the outside of the ring (Figure 4C).

After we obtained the two predicted distinct conformational states of the DndCDE complex structure (Supplementary Figure S6), we further used Chai-1 to predict two distinct conformational states of *E. coli* B7A DndCDE in a complex with 23 base pairs (bp) of DNA (Figure 4D,F and Supplementary Table S2). Interestingly, the DNA inserts into the hollow hole formed by the predicted DndCDE complex, with the three proteins DndC, DndD, and DndE wrapping surrounding it. The cylindrical shape of DNA is complementary to that of the hollow cavity of the predicted DndCDE complex, with DndC and DndE forming close contact with DNA (Figure 4G). Our normal mode analysis (NMA) [36] results confirmed our hypothesis that conformational transition enables the predicted DndCDE complex to cycle between association with DNA and dissociation from it (Figure 4H,I and Supplementary Movie S1). Presumably, the closed conformation allows the predicted DndCDE complex to grasp the DNA, and the open conformation allows the predicted DndCDE complex to release the PT-modified DNA; thus, it can enter into another cycle of DNA PT modification.

### 2.6. Recognition of the Core Consensus Phosphorothioation Motif by DndC

**Figure 5:**
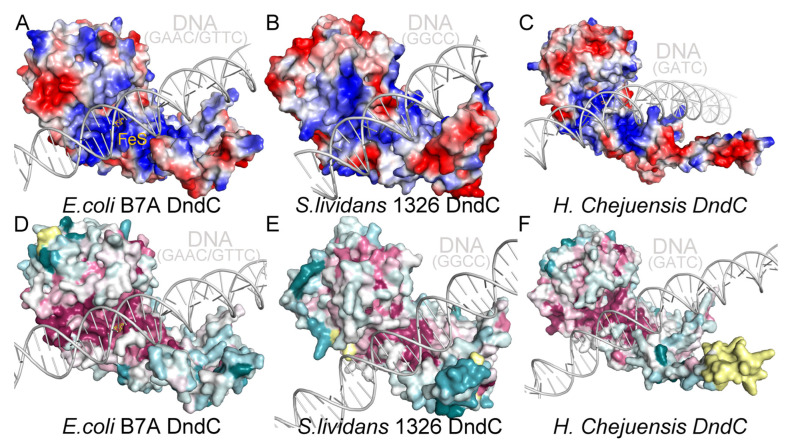
DndC is predicted to employ a highly conserved, positively charged groove for DNA recognition. (**A**–**C**) DNA with GAAC/GTTC, GGCC/GGCC, and GATC/GATC repeat sequences are predicted to bind within the positively charged groove of *E. coli* B7A (**A**), *S. lividans* 1326 (**B**), and *H. Chejuensis* KCTC2396 DndC (**C**), respectively. (**D**–**F**) A computational analysis predicts that the surfaces of the DNA-binding grooves of *E. coli* B7A (**D**), *S. lividans* 1326 (**E**), and *H. Chejuensis* KCTC2396 DndC (**F**) are highly conserved.

We used Chai-1 to predict the structure of a fragment of DNA with different core consensus phosphorothioation motifs in a complex with their corresponding DndC (for example, GAAC/GTTC with *E. coli* B7A DndC, GGCC/GGCC with *S. lividans* 1326 DndC, and GATC/GATC with *H. Chejuensis* KCTC2396 DndC) equipped with the iron–sulfur cluster. We found that, in each case, DNA is harbored in a positively charged (Figure 5A–C) and highly conserved groove on the surface of DndC (Figure 5D,F).

**Figure 6:**
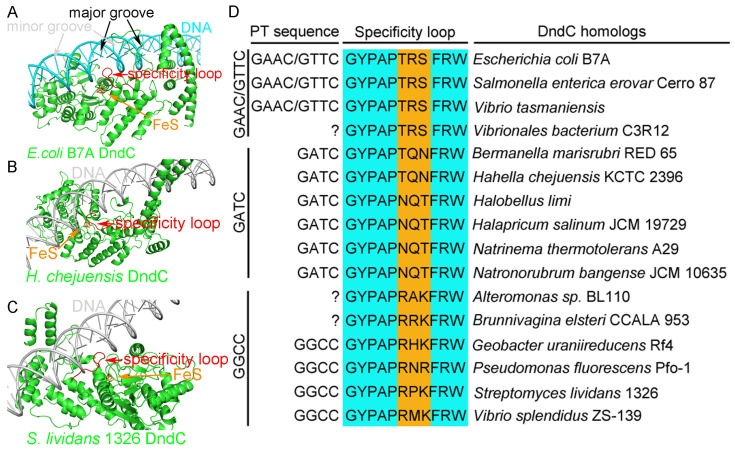
The specificity loop of DndC is predicted to recognize the core consensus phosphorothioation motif on DNA. The black arrow points to the DNA’s major groove, the gray arrow indicates the minor groove, the red arrow highlights the specificity loop, and the orange arrow marks the iron–sulfur cluster. (**A**) The specificity loop of *E. coli* B7A DndC is predicted to insert into the major groove of DNA and interact with the GAAC/GTTC core consensus phosphorothioation motif. (**B**) The specificity loop of *H. chejuensis* KCTC2396 DndC is predicted to insert into the major groove of DNA with the GATC sequence motif. (**C**) The specificity loop of *S. lividans* 1326 DndC is predicted to insert into the major groove of DNA with the GGCC sequence motif. (**D**) Three positions on the conserved specificity loop display subtype-specific conservation with respect to the three kinds of DNA PT modification. “?”indicates that the PT modification status is yet unknown.

More importantly, a loop on DndC (e.g., G^135^YPAPTRSFRW^145^ of *E. coli* B7A DndC), which is close to the iron–sulfur cluster, was predicted to insert into the major groove of DNA and form sequence-specific interactions with the bases of the core consensus phosphorothioation motif (Figure 6A). This phenomenon is not only predicted in *E. coli* B7ADndC, but also in *H. chejuensis* KCTC2396 DndC (Figure 6B) and *S. lividans* 1326 DndC (Figure 6C).

Three positions on this conserved specificity loop, i.e., residues 140–142 for *E. coli* B7A DndC, seemingly display subtype-specific conservation with respect to the three kinds of DNA PT modification. In *E. coli* B7A, *Salmonella enterica* Cerro 87, and *Vibrio tasmaniensis* 1F-267, DNA PT exists on the G_PT_AAC/G_PT_TTC consensus motif, and the residues at these three positions on the conserved specificity loop of DndC are unanimously Thr-Arg-Ser (e.g., T^140^R^141^S^142^ for *E. coli* B7A DndC). On the other hand, in *Hahella chejuensis* KCTC2396, *Bermanella marisrubri* RED65, *Halobellus limi*, *Halapricum salinum* JCM 19729, *Natrinema thermotolerans* A29, and *Natronorubrum bangense* JCM 10635, whose DNA PT core consensus motif is G_PT_ATC, these three positions of DndCʹs conserved specificity loop harbor Thr-Gln-Asn residues or Asn-Gln-Thr residues (e.g., T^138^Q^139^N^140^ for *H. chejuensis* KCTC2396 DndC and N^138^Q^139^T^140^ for *Halobellus limi* DndC). Lastly, in *Streptomyces lividans* 1326, *Vibrio splendidus* ZS-139, *Pseudomonas fluorescens* Pf0-1, and *Geobacter uraniumreduces* Rf4, DNA PT occurs on the G_PT_GCC consensus motif, and the three positions on the conserved specificity loop of DndC from these bacteria have an Arg-Xxx-Lys/Arg pattern (e.g., R^132^P^133^K^134^ for *S. lividans* 1326 DndC) (Figure 6D and Supplementary Figure S7).

### 2.7. Recognition of DNA by DndE

**Figure 7:**
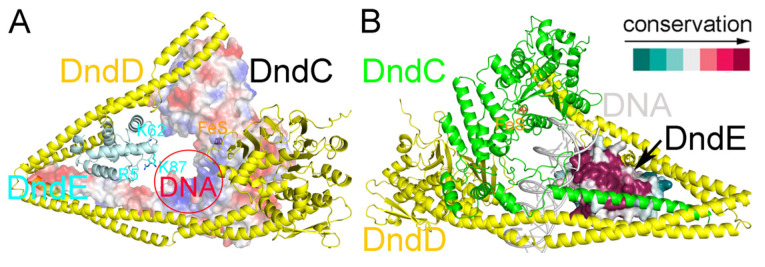
Structural modeling predicts DNA recognition by DndE. (**A**) Three positively charged residues of *E. coli* B7A DndE are predicted to point toward the hollow space where the bound DNA might be located. (**B**) The predicted DNA-binding surface of DndE is highly conserved.

In our predicted structure of the *E. coli* B7A DndCDE complex, three positively charged residues of DndE, R5, K62, and K87 point toward the inner hollow space where the bound DNA might be located (Figure 7A). In addition, these residues of *E. coli* B7A DndE speculated to be involved in DNA binding are highly conserved. This can be seen from the Chai-1 predicted structure of *E. coli* B7A DndCDE-DNA complex, in which the DNA-binding surface of DndE is highly conserved (Figure 7B). It is envisaged that there exists an interplay among the three subunits of the DndCDE complex during the DNA binding and PT modification processes.

### 2.8. Microbiological Assays Showing the Importance of the Specificity Loop in DNA PT

**Figure 8:**
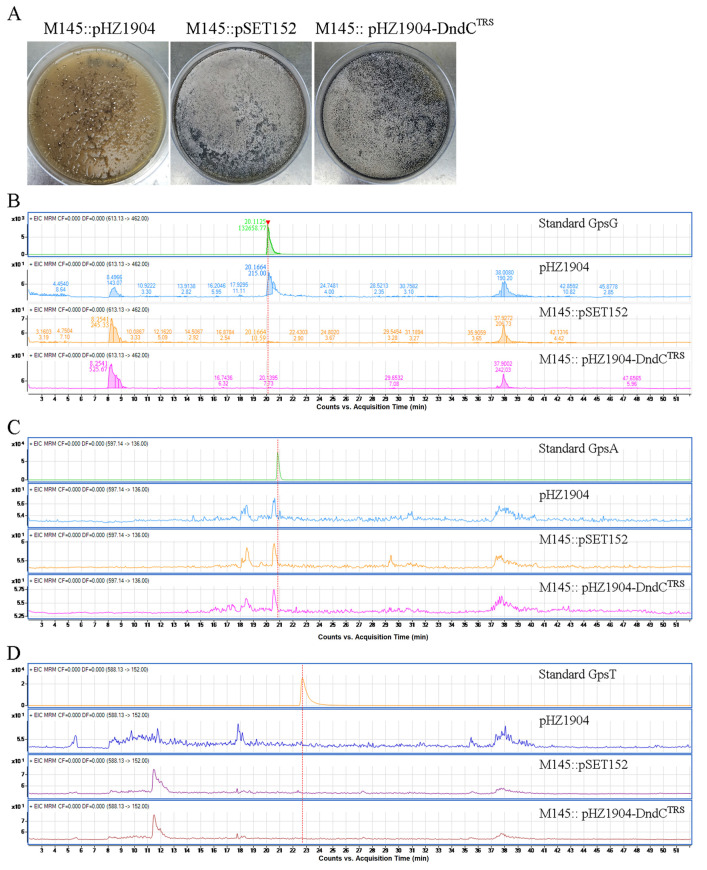
A conjugative transfer and an LC-LS assay supported the key role of the specificity loop of DndC in DNA PT. (**A**) The conjugative transfer between *Streptomyces coelicolor* M145 and *E. coli* with pHZ1904 plasmid did not occur (the left dish). The M145::pSET152 exconjugants and M145::pHZ1904-DndC^TRS^ exconjugants grew normally (the middle and right dishes, respectively). (**B**–**D**) Using the LC-MS assay to examine the G_PT_G (**B**), G_PT_A (**C**), and G_PT_T (**D**) modification, which comes from standard, pHZ1904, M145::pSET152 exconjugants, and M145::pHZ1904-DndC^TRS^ exconjugants. For positive control, the G_PT_G, G_PT_A, and G_PT_T modification peaks from the standard and the G_PT_G modification peak from pHZ1904 plasmid were observed. However, G_PT_G, G_PT_A, and G_PT_T modification peaks from M145::pSET152 exconjugants and M145::pHZ1904-DndC^TRS^ exconjugants were not detected. (M145 means *Streptomyces coelicolor* M145.) The dotted lines indicate the peak positions of the standard samples.

In order to investigate whether the specificity loop of DndC indeed plays a key role in DNA PT modification in vivo, we performed conjugative transfer and liquid chromatography–mass spectrometry (LC-MS) assays using DNA extracted from bacteria transformed with an empty vector, wild type DndC, or mutant DndC. As a negative control, the conjugative transfer between *Streptomyces coelicolor* M145 and *E. coli* with pHZ1904 plasmid did not occur because ScoMcrA expressed from *Streptomyces coelicolor* M145 cleaves DNA sequences containing G_PT_GCC modifications in the pHZ1904 plasmid (Figure 8A). In contrast, the conjugants carrying DNA from *Streptomyces coelicolor* M145 and the pSET152 plasmid grew very well. Meanwhile, the conjugants carrying DNA from *Streptomyces coelicolor* M145 and the pHZ1904-DndC^R132T/P133R/K134S^ (pHZ1904-DndC^TRS^) plasmid also grew very well. This result demonstrates that the mutation of the three core amino acids in the specificity loop caused DndC to lose the PT modification capability on the GGCC consensus sequence. Furthermore, our LC-MS assays also showed that the mutation of the three core amino acids in the specificity loop in DndC from other bacteria also caused DndC to lose the G_PT_G, G_PT_A, or G_PT_T modification capability (Figure 8B–D). This suggests that the three core amino acids in the specificity loop of DndC play a key role in the PT modification process. The particular mechanism of how these three core amino acids participate in DNA PT modification is still being investigated.

## 3. Discussion

DNA PT is the only epigenetic modification that has been found to occur on the DNA backbone so far, which makes it a unique phenomenon to study. However, until now we still do not understand clearly the structure and the DNA recognition mechanism of the central player in DNA PT, the DndCDE protein complex. By using AlphaFold3 and Chai-1, we obtained high-confidence predicted structures of DndC, DndD, the DndC-DndD complex, the DndD-DndE complex, the DndCDE complex, and the DndCDE-DNA complex.

Although AlphaFold3 performs excellently in predicting the structures of monomeric proteins and static protein–protein complexes, existing studies have revealed its limitations in modeling systems involving complex RNA tertiary structures [37], non-canonical RNA conformations, or multiple metal-binding sites [38]. For example, Bernard et al. pointed out that AlphaFold3 often simplifies or misfolds large RNA molecules due to its limited understanding of long-range base-pairing interactions. Similarly, Krokidis et al. emphasized its inability to accurately capture conformational flexibility in dynamic RNA systems [39]. Therefore, while we use AlphaFold3 to predict the overall structure of the DndCDE complex, we also recognize these limitations and highlight the need for complementary tools.

To address these shortcomings, particularly in capturing detailed molecular interactions such as ligand binding, we also employed Chai-1. Abramson et al. demonstrated that Chai-1 outperforms AlphaFold3 and RoseTTAFold All-Atom in modeling small-molecule binding sites, accurately simulating key non-covalent interactions such as hydrogen bonds, hydrophobic effects, and π–π stacking [40]. However, Chai-1 also has its own drawbacks: although it can predict accurate single-chain conformations, it sometimes fails to determine the correct relative orientations between chains. Therefore, our strategy is to combine the strengths of both methods—using AlphaFold3 for monomer or overall complex prediction, while employing Chai-1 to refine local interaction details, especially near the 4Fe-4S or DNA binding interfaces.

We propose that by alternating between “closed” and “open” conformations, the DndCDE complex cycles between association with and dissociation from DNA, so that it can introduce PT modification into multiple core consensus sites on DNA. Our predicted interaction interface between DndC and DndD and that between DndD and DndE were supported by a protein surface conservation analysis and were further verified by in vitro biochemical pull-down assays using point mutant proteins.

It has been found that there are three kinds of core DNA PT consensus sequences for the DndCDE complex, G_PT_AAC/G_PT_TTC, G_PT_GCC, and G_PT_ATC. However, it has not been understood how DNA sequence-specific PT modifications occur. In this work, we revealed that a conserved loop, referred to as the specificity loop, on DndC played a key role in DNA PT modification. Three core amino acids were found to be especially important. These three residues on the specificity loop apparently provide sequence specificity with respect to the three kinds of DNA PT modification. A rough pattern could be concluded that these three positions are “Thr-Arg-Ser” for the G_PT_AAC/G_PT_TTC modification, “Arg-Xxx-Lys/Arg” for the G_PT_GCC modification, and “Thr-Gln-Asn” or ”Asn-Gln-Thr” for the G_PT_ATC modification. Therefore, it may imply that by looking at what the residues are at these three positions of the specificity loop of DndC, we would be able to judge what kind of PT modification occurs in the particular bacteria from which the DndC protein is derived.

In summary, AlphaFold3 and Chai-1 offer powerful tools for structure prediction, each with distinct advantages and limitations. Although these technologies represent the current state of the art and some comparative studies have been published, their unique strengths and potential weaknesses in practical applications remain incompletely understood [41]. AlphaFold3 excels at predicting stable protein folds and complex architectures, while Chai-1 performs particularly well in modeling detailed molecular interactions involving ligand binding. It is important to note that all structures to date are predicted, and their accuracy may be limited by inherent biases in the methods themselves. Moreover, despite structural biologists’ efforts to ensure the correctness of published structures, errors can still occur in both predicted and experimentally determined structures and may sometimes be difficult to detect or correct [42]. Therefore, integrating these computational approaches with a rigorous experimental validation remains an essential path toward achieving a comprehensive mechanistic understanding of biological processes such as DNA phosphorothioation modification mediated by the DndCDE complex.

## 4. Materials and Methods

### 4.1. Protein Structure Prediction

We utilized AlphaFold3 (https://alphafoldserver.com/ accessed on 2 February 2025) and Chai-1 (https://lab.chaidiscovery.com/dashboard accessed on 2 February 2025) with full multiple sequence alignment (MSA) information to predict the structures of various biological complexes. Specifically, AlphaFold3 was employed for predicting the structures of individual proteins or protein-protein complexes, while Chai-1 was primarily used for predicting complexes involving ligands or DNA sequences. The process began with the preparation of input data, which included protein sequences in FASTA format, DNA sequences, or ligand SMILES representations. These inputs were then fed into the respective tool—AlphaFold3 for single proteins and protein–protein interactions and into Chai-1 for modeling more complex systems such as protein–ligand or protein–DNA–ligand interactions. The modeling workflow generated five predicted structures for each target complex, ranging from simple binary interactions to more complex multi-component systems involving proteins, DNA, and ligands. The accuracy of the predicted structures was evaluated using metrics such as pTM (predicted Template Modeling score) and ipTM (interface Template Modeling score). Finally, the results were visualized using PyMOL (version 2.3) [28,31,32]. The Dali server was used to find the closest structural homologue of DndC, from which APS reductase was chosen by checking the Z-score. All DNA sequences are provided in Supplementary Table S2. The protein surface conservation was analyzed by the Consurf server [43]. All the DndC/DndD/DndE proteins studied in this work belong to *Escherichia coli* B7A unless otherwise indicated.

### 4.2. Protein Expression and Purification

All the DndC and DndE constructs were subcloned into the pACYCDuet plasmid with 6×His-tags. All the DndD constructs were subcloned into the pETDuet plasmid with Flag-tags. The plasmids were transformed into *E. coli* BL21 (DE3) competent cells and cultured at 37 °C until OD_600_ at 0.6 and then induced by 0.2 mM IPTG and cultured at 16 °C for 18 h.

The cells were lysed with a high-pressure homogenizer (Union-Biotech, Shanghai, China) after harvesting and resuspension. After centrifugation at 14,000 rpm for 1 h, the supernatant was subjected toNi^2+^-NTA or anti-Flag affinity chromatography. After extensive washing by the washing buffer (50 mM Tris-HCl, pH 8.0, 150 mM NaCl, and 5% glycerol), the proteins were eluted with the elution buffer (50 mM Tris-HCl, pH 8.0, 150 mM NaCl, 5% glycerol, 500 mM imidazole or 200 ng/μL Flag peptide) and then analyzed by SDS-PAGE and Coomassie blue staining.

### 4.3. Electron Microscopy

The DndC-DndD protein complex or the DndCDE protein complex purified above was further purified through the HiLoad 16/600 Superdex 200 pg gel filtration chromatography (GE Healthcare, Chicago, IL, USA). The equilibration buffer used was 50 mM Tris-HCl, pH 8.0, 150 mM NaCl, and 5% glycerol. Peak fractions were collected. Then, 5 μL protein samples were adsorbed for 60 s onto carbon-coated copper grids, which were treated for 60 s by a glow discharger at room temperature. Excess buffer on the grids was taken away by a filter paper, washed with 2% uranium acetate (UA), and negatively stained with 2% UA for 60 s. All micrographs were collected using an FEI TF20 TEM at a 62,000-fold magnification, an acceleration voltage of 200 kV, and a total dose of 20 e^−^/Å^2^ on a 4K×4K CCD camera. All micrographs were processed with cryoSPARC, including CTF estimation, particle picking, and 2D classification. For the sample of the DndC-DndD complex, 36,644 particles were aligned and classified into 51 classes. For the sample of the DndCDE complex, 8634 particles were aligned and classified into 30 classes.

### 4.4. Conjugative Transfer [44]

*E. coli* ET12567/pUZ8002 (kanamycin- and chloramphenicol-resistant) and *Streptomyces coelicolor* M145 strains were grown in LB and SFM mediums, respectively, supplemented with 50 μg/mL kanamycin or 50 μg/mL chloramphenicol or made antibiotic-free according to requirement. The transformation of donor *E. coli* ET12567/pUZ8002 with the empty vector pSET152 (apramycin-resistant), pSET152::*dnd*A-*dnd*E (named pHZ1904, apramycin-resistant), or pHZ1904 carring DndC^R132T/P133R/K134S^, whose *dnd*A-*dnd*E was derived from *Streptomyces lividans* 1326, was performed using the Easyject Plus electroporator (BIO-Rad, Hercules, CA, USA). Exconjugants were selected and cultured overnight at 37 °C. The overnight culture was then inoculated into a fresh LB medium at a 1:10 ratio and incubated at 37 °C until OD_600_ = 0.6. The bacterial cells were collected by centrifugation at 8000 rpm for 10 min and washed three times with fresh LB to remove residual antibiotics. Spores of the recipient strain *Streptomyces coelicolor* M145, including a type IV restriction enzyme (ScoMcrA) that recognizes and restricts the DNA sequence G_PT_GCC/G_PT_GCC but not G_PT_AAC/G_PT_TTC, were suspended in 1 mL of LB medium, followed by two times of washing with TES buffer. Then, 500 μL of TES buffer was added, and the mixture was heat-shocked at 50 °C for 10 min. Subsequently, 500 μL of a 2×YT medium was added, and the culture was incubated at 30 °C for 2 h. The donor and recipient strains were mixed (200 μL each) by gentle pipeting. The mixture was spread on SFM plates without antibiotics, air-dried, and incubated at 30 °C for 14–16 h. The plates were covered with the 30 μg/mL apramycin and 0.5 mg/mL nalidixic acid; after 3–4 days, the conjugants could be observed. During conjugation transfer, ScoMcrA cleaves gene sequences containing G_PT_GCC modifications. Therefore, the absence or a low quantity of conjugants indicates the presence of genes with G_PT_GCC/G_PT_GCC modifications, thereby confirming the DNA PT modification capability of DndC. Conversely, if conjugants are present and their quantity is high, it demonstrates that DndC lacks sulfur modification capability.

### 4.5. Using LC-MS to Examine Bacterial DNA PT Modification [21]

Bacteria (10 mL) were centrifuged and re-suspended with 500 μL of a TE buffer. Then, 20 μL of lysozyme (2 mg/mL) was added and then warmed at 37 °C for 20 min. A total of 50 μL of 10% SDS, 25 μL of protease K (20 μg/μL), and 10 μL of RNaseA (2 mg/mL) were added and mixed gently. The mixture was heated at 55 °C for 30 min and mixed gently every 10 min. Phenolic chloroform (600 μL) was added under the fume hood. The mixture was shaken vigorously until becoming milky white, and it was centrifuged at 14,000× *g* for 10 min. Then, 500 μL of the supernatant was transferred to an empty eppendorf tube, where 50 μL of 3 M sodium acetate was added and shaken well. Isopropyl alcohol (385 μL) was added and mixed gently upside down to precipitate DNA. DNA was removed from the liquid and transferred to a new tube containing 500 μL of 70% iced ethanol and then centrifuged at 12,000× *g* for 10 min at 4 °C. The supernatant was discarded, and the precipitate was washed with 70% iced ethanol. After another centrifugation, the precipitate was air-dried. Then, 300 μL of ddH_2_O was added, heated at 50 °C for 30 min, put in 4 °C refrigerator for 30 min, and then stored in −40 °C freezer.

Genomic DNA (20 μg) was taken and denatured at 90 °C for 3 min. Then, it was immediately put on ice for 15 min to cool down. Genome DNA (20 μg), 2 μL of NP1, 1 μL of 3 M sodium acetate, and 1 μL of zinc chloride were mixed, and ddH_2_O was added to reach a total volume of 100 μL, and the mixture was heated at 50 °C for 2 h. Then, 100 μL of genome DNA, 10 μL of 1 M Tris-HCl, and 2 μL of CIP were mixed and heated at 37 °C for 4 h.

For the preparation of the mass spectrometry sample, the column was washed with 400 μL of ddH_2_O and centrifuged at 10,000× *g* for 15 min. The supernatant was discarded, and all of the reaction system was added onto the filter membrane. ddH_2_O (100 μL) was added to wash the tube and was also transferred to the filter membrane. It was centrifuged at 10,000× *g* for 15 min. Then, 200 μL of ddH_2_O was added to the filter membrane, and it was centrifuged at 10,000× *g* for 15 min to wash the residual bases on the filter membrane. After that, 22 μL of ddH_2_Owas added and centrifuged at 10,000× *g* for 15 min. Then, 20 μL of a sample including different standard samples was added to the inner tube of the sample bottle for mass spectrometry.

## Data Availability

No additional data are available for this manuscript. All relevant data are included within the article and its supplementary files.

## Supplementary Materials

The following supporting information can be downloaded at: https://www.mdpi.com/article/10.3390/ijms26125765/s1.
