# Supplementary material for: Molecular Insight into the Recognition of DNA by the DndCDE Complex in DNA Phosphorothioation
Source: Int J Mol Sci. 2025 Jun 16;26(12):5765. doi: 10.3390/ijms26125765 (PMC12193691; doi:10.3390/ijms26125765)
Supplement: Supplementary file 1 [file ijms-26-05765-s001.zip › Supplementary Information.pdf]

Supplementary Information

Molecular insight into the recognition of DNA by the DndCDE complex in DNA phosphorothioation

Wencheng Fu[1], Yuli Wang[1], Yashi Ge[2], Haiyan Gao[2], Xuan Sun[1], Zixin Deng[1], Lianrong Wang[2], Shi Chen[2], Xinyi He[1], Geng Wu[1]*

[1]State Key Laboratory of Microbial Metabolism, School of Life Sciences & Biotechnology, the Joint International Research Laboratory of Metabolic & Developmental Sciences MOE, Shanghai Jiao Tong University, Shanghai 200240, China

[2]Key Laboratory of Combinatorial Biosynthesis and Drug Discovery, Ministry of Education, School of Pharmaceutical Sciences, Wuhan University, Wuhan, China

*To whom correspondence may be addressed: geng.wu@sjtu.edu.cn

Supplementary Table:

Supplementary Table S1:

The presentation of pTM scores within the proteins, as well as the ipTM + pTM quality metrics for each protein pair. pTM and ipTM scores: The predicted template modeling (pTM) score and the interface predicted template modeling (ipTM) score are both based on a metric known as the template modeling (TM) score. This metric evaluates the overall accuracy of the predicted structure. A pTM score exceeding 0.5 suggests that the overall fold of the complex may resemble the actual structure. The ipTM score, on the other hand, assesses how accurately the relative positions of subunits within the complex have been predicted. Scores above 0.8 indicate high confidence in the prediction, whereas values below 0.6 suggest a likely failure in predicting the correct interface. ipTM scores falling between 0.6 and 0.8 represent an uncertain range, where the prediction could either be correct or incorrect.

	**AF3**			**Chai-1**		
**Structure**	**ipTM score**	**pTM score**	**ipTM+ pTM score**	**ipTM score**	**pTM score**	**ipTM+ pTM score**
DndC	\	0.86	\	\	\	\
DndC-FeS（rank0）	\	\	\	0.22	0.88	1.1
DndC-FeS（rank3）	\	\	\	0.21	0.88	1.09
DndD	\	0.67	\	\	\	\
DndD dimer-DNA	\	\	\	0.49	0.62	1.11

DndCD	0.67	0.69	1.36	\	\	\
DndDE	0.88	0.37	1.25	\	\	\
DndCDE (rank0)	0.52	0.61	1.13	\	\	\
DndCDE (rank1)	0.52	0.59	1.11	\	\	\
DndCDE-DNA (rank0)	\	\	\	0.55	0.71	1.26
DndCDE-DNA (rank1)	\	\	\	0.55	0.71	1.26
Dnd D globular domain dimer-DNA-ATPγS complex	\	\	\	0.75	0.81	1.56

Supplementary Table S2:

Summary of all DNA sequences analyzed in this study.

DNA		Sequence (5'-3')
DndD dimer-DNA	sense	CCTCTTGCGGGAACTCTCGCTTTG
	antisense	CAAAGCGAGAGTTCCCGCAAGAG
ATPγS-bound DndD Globular Domain Dimer-DNA Complex	sense	CCTCTTGCGGGAACTCTCGCTTTG
	antisense	CAAAGCGAGAGTTCCCGCAAGAG
DndCDE-DNA	sense	CCTCTTGCGGGAACTCTCGCTTTG
	antisense	CAAAGCGAGAGTTCCCGCAAGAG
E.coli B7A DndC-DNA	sense	GAACGAACGAACGAACGAACGAACGAACGAAC GAACGAACGAACGAACG
	antisense	CGTTCGTTCGTTCGTTCGTTCGTTCGTTCGTTCGT TCGTTCGTTCGTTC
S.lividans 1326 DndC-DNA	sense	GGCCGGCCGGCCGGCCGGCCGGCCGGCCGGCC GGCCGGCCGGCCGGCCG
	antisense	CGGCCGGCCGGCCGGCCGGCCGGCCGGCCGGCC GGCCGGCCGGCCGGCC
H.Chejuensis	sense	GATCGATCGATCGATCGATCGATCGATCGATCGAT

DndC-DNA		CGATCGATCGATCG
	antisense	CGATCGATCGATCGATCGATCGATCGATCGATCGA<br>TCGATCGATCGATC

Supplementary Figures:

Supplementary Figure S1.

Structural comparisons of DndC and APS crystal structures predicted by Chai-1 and AlphaFold3 (AF3). (A) The Chai-1-predicted DndC model (cyan) exhibited strong agreement with the AF3-predicted DndC (magenta) in core regions, as evidenced by a low RMSD of 0.781. (B) Furthermore, spatial proximity was observed between the Chai-1-predicted 4Fe-4S cluster (orange) in DndC and the crystallographically resolved 4Fe-4S cluster (green) in APS reductase (green; PDB: 2GOY), with an alignment RMSD of 3.926. (C) The Chai-1 prediction of DndC revealed two distinct conformational states in the loop region (residues 256–279). The rank0 model (green, ipTM+pTM=1.10) adopts a closed conformation, while the rank3 model (yellow, ipTM+pTM=1.09) displays an open state.

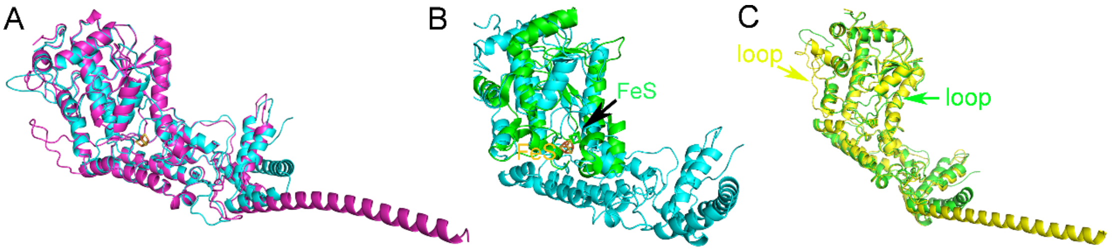

Supplementary Figure S2.

Structural alignment of Chai-1-predicted DndD globular domain dimer-DNA-ATPγS with the experimentally determined Rad50-DNA-ATPγS （PDB：5DNY）architecture. Color coding: green represents the ATPγS-bound DndD globular domain dimer-DNA complex; red denotes the Rad50-DNA-ATPγS complex. Panels (A), (B), and (C) display front, side, and top views, respectively, while Panel (D) illustrates ligand alignment.

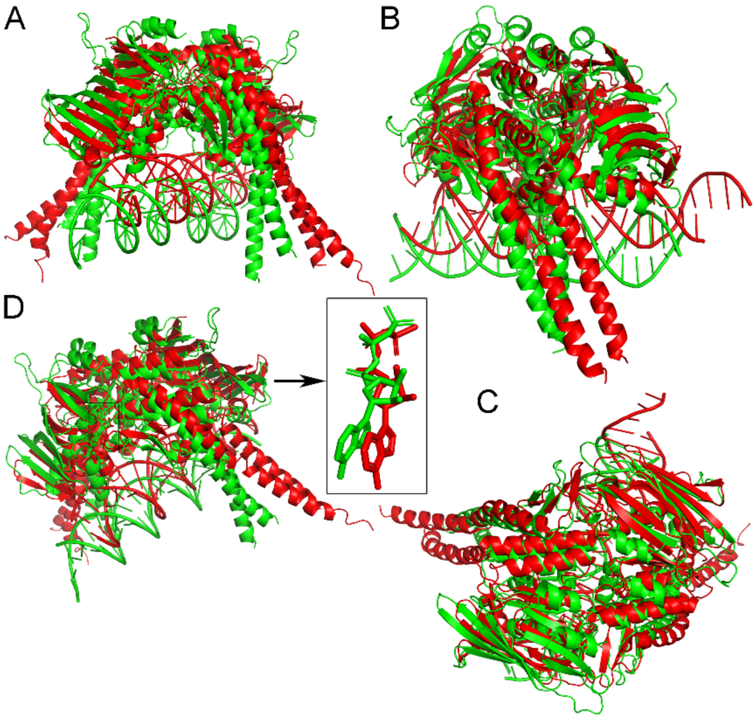

Supplementary Figure S3.

2D classification result of the negative staining electron microscopy images of the *E. coli* B7A DndC-DndD protein complex. Green circles denote the putative complex of DndC and the globular domain of DndD. Red arrows denote the putative coiled coil domains of DndD

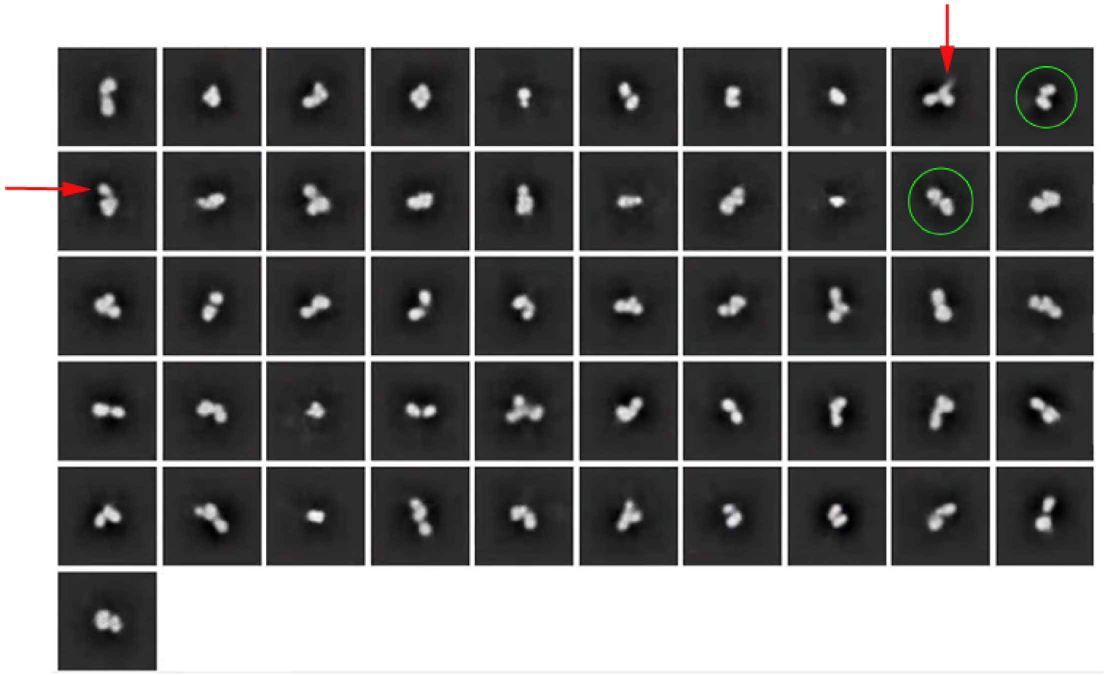

Supplementary Figure S4.

Nickel column pull down assays using purified proteins confirmed that DndC binds to DndD with coiled coil-2 deleted or DndD with both coiled coil-1 and -2 deleted. (**A**) DndC binds to DndD with coiled coil-2 deleted. (**B**) DndC binds to DndD with both coiled coil-1 and -2 deleted. Note: In the two distinct DndD coiled-coil deletion mutants, each deleted segment is replaced with a glycine-serine linker (GSGSGSGSGS) to link the rest of fragments.

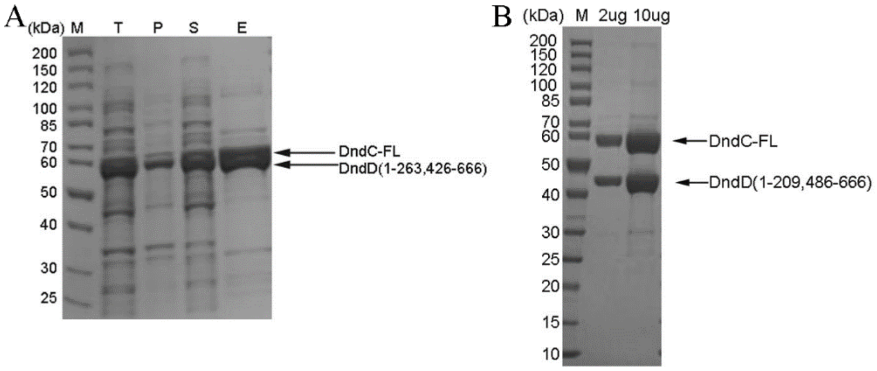

Supplementary Figure S5.

2D classification result of the negative staining electron microscopy images of the *E. coli* B7A DndCDE protein complex. Yellow circle denotes putative dimer of the DndCDE complex resulting from dimerization of DndD.

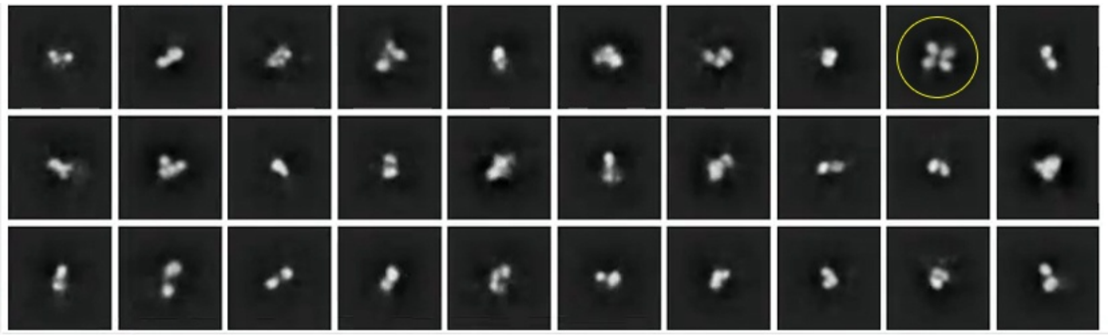

Supplementary Figure S6.

The two distinct conformational states of the DndCDE structure predicted by AF3 are colored green (State 1, rank0) and red (State 2, rank1).

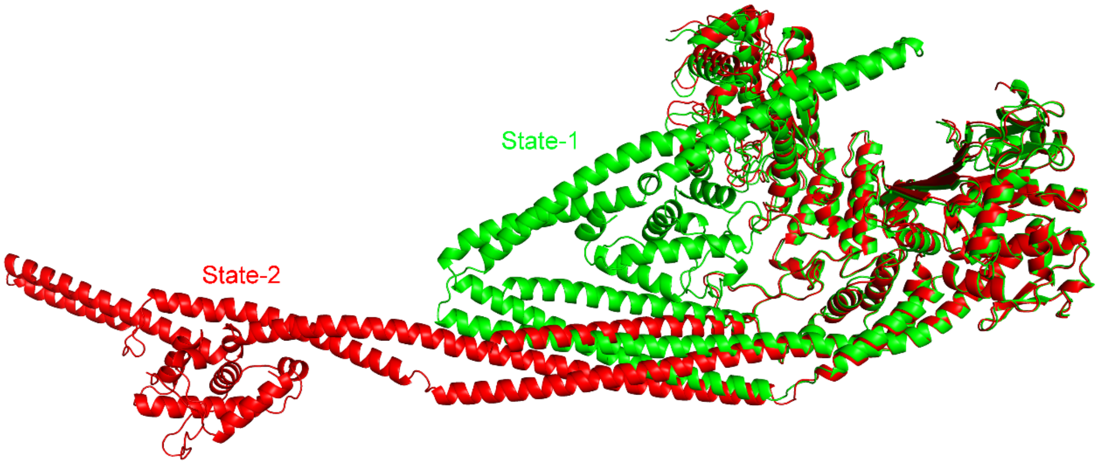

Supplementary Figure S7.

Multiple sequence alignment of DndC. The protein alignment reveals a highly conserved specificity loop (highlighted by red frame) across all 16 DndC homologs, with the exception of three core residues (marked in orange frame). The height of the colored columns corresponds to the consistency intensity of amino acids.

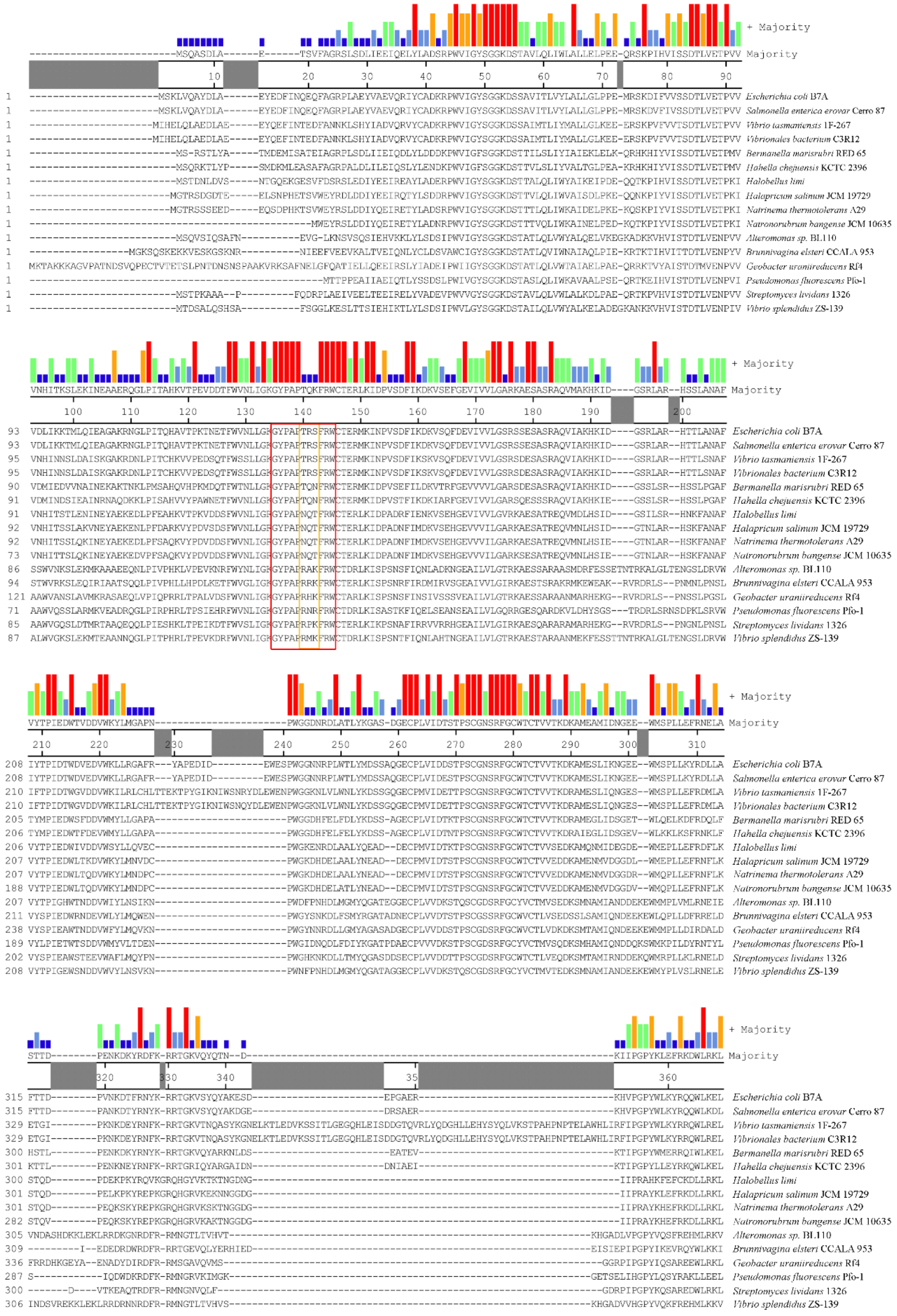

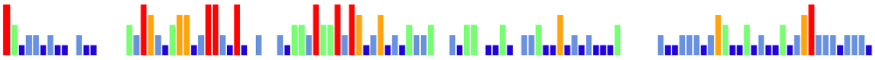

```
                                                                                                                        + Majority
    LETEKEVNELGPD----IELITDEELHAIRRIWLTD--EPDWEDSLPKIYNEVYGEDLDW--VRDDLGSFGAEDADLLKELCAEFD-----VPPELVMKLLDLELQMRGLSRRAGIYNKLD Majority
    370       380           390       400       410       420          430       440            450       460       470
370 LEIERDLNAQGHT----ITLITKPELHAIRQEWLKDPNEPDWYDTLPGIYREVYQQDLDW--VVDDQSRFDASDADLLVQIAQGFD-----VVPEMVMKLIELEVSMEGLSRRQGIFDKLG Escherichia coli B7A
370 LEIERDLNAQGHT----ITLITQPELHAIRQEWLKDPNEPDWHDTLPEIYREVYQQDLNW--VVDDQSRFDASDADLLAQITQGFD-----VVPEMVMKLIELETSMEGLSRRQGIFDKLG Salmonella enterica erovar Cerro 87
441 LELDKKYKSEGRE----IELITVPELHAIRQEWIHDPNEPDWDDSLPTIFNEVYGYDLDW--VYDDNASFGKDDALIINELSEDFD-----IAPEMIMKLIELEVSMEGLSRRSGINNKIA Vibrio tasmaniensis 1F-267
441 LELDKKYKSEGRE----IELITVPELHAIRQEWIHDPNEPDWDDSLPTIFNEVYGYDLDW--VYDDNASFGKDDALIINELSEDFD-----IAPEMIMKLIELEVSMEGLSRRSGINNKIS Vibrionales bacterium C3R12
354 LTIEKKLRDSGKD----ITLIHPDELYAIRREWMYDPNEPDWADTAPKIYREVYGKDLDW--ADQDAGSFSQSDAEILSSLGAEFD-----VPSELVMKLLDLEQAMDGLSRRKGLYKKID Bermanella marisrubri RED 65
356 LTIEKNLIEEGRK----IQLIHPEELHAIRKEWMFDPNEPDWSDELPKIYHSVYGEDLDW--VEHDSGAFSQADADLIAELSAENG-----VPAELVMKLLELELSMDGLSRRKGLFNKIE Hahella chejuensis KCTC 2396
349 LETQKEVNEQIPDDEE-MDLIRDEELREIRRIWREE--RADWEDSVPKIYREAMEEDIGW--VQDDYGAFGETEAKLLKEIADKHD-----VPPELVQRLLDTELQHHGMKRRASIYNEID Halobellus limi
350 LETEKEVNELRDDDAEYLDLIRDEELKEIRRLWRTE--EADWEDSVPKIYNEVMDDELDW--VRDDLGSFSTEEAKVLERVCEKHD-----VPDKLVKRLLDTELQHHGMKRRASIYNEID Halapricum salinum JCM 19729
350 LETEKEVNELRDDDAEYLDLIRDEELKEIRRLWRTE--EADWEDSVPKIYNDVMDDDLDW--VRDDLGSFSTEEAEVLKQVCAEHD-----VPEKLVKRLLDTELQHHGMKRRAAIYNKID Natrinema thermotolerans A29
331 LETEKEVNELREDNEEYLDLIRDEELKEIRRLWRTE--EADWEDSVPKIYNDVMDDDLDW--VHDDLGSFSTEEAEVLEEVCAQYD-----VPEKLVKRLLDTELQHHGMKRRAAIYNKID Natronorubrum bangense JCM 10635
362 LEAQVAVQQMAPAEVNDLELISIEELEGIREVWLND--KHEAEDSLPLIYEQVIGKAYPG-KRRAHHPIINKSVLDKLKAHCAEQGDEDG-LLYQQMRAVLNVSNKYRNQLRRAKLKDELN Alteromonas sp. BL110
359 LAVERRLRITAPEGMRDITLITREELSEIRHIWLNE--RHEFDDSLPRIYKEVTGEEFIDPRPGADASLLGSDEWSVLEEICGGDA-----MHLELMAKLLDTEYQFRKRSRRVGIYDALE Brunnivagina elsteri CCALA 953
388 LIAQTYIRKNGPPEVQGIELITLEELQEIRRIWVVD--KHELEDSLPRIYREATGNDYPG-RPLDDNLVIGEEEMHVLAELCGEDR-----LHYELTRELLSITRQQRTTSRRAGLHEQLE Geobacter uraniireducens Rf4
332 LRTQLTLRNAKIPGHDQIELISIEEIDAIRRIWVEE--KGEIEDLVPKIYERVLQQSYPG-QAMEQHPLE-PEDLALLNDVANEVDSEAGGRLYQLARNLLAVQYQSMQSQKRSHHLDRLE Pseudomonas fluorescens Pfo-1
342 LAAQAQVRKKAPEHVRNIELISMAELQEIRRIWVFE--KHEVEDSLPQIYEQATGEKFPG-LPLDEQLVLGAEEIGLLKEACEDDP-----IHFSMTRELLAIERQYRTMTRRAGLFEALE Streptomyces lividans 1326
363 LEAQVAVQHMGPPEVKDLELLTLEDLEAIRKIWLED--KHEIEDNLPKIYEQVIKQPYEG-KRRAHHPILNSESLSKLQTYCEQHGDKEG-LLYQQIRATMSVANKFRSQLRRAKLGEELN Vibrio splendidus ZS-139
```

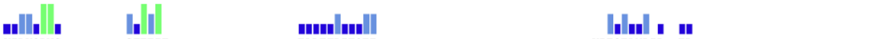

```
                                                                                                                        + Majority
    KILKQDWG---------SFEEIT------------------EALEFDIEQEE----------------------------KPEQELYDID--EL---------------------- Majority
    480                490                 500       510                               520       530       540
480 TILKQDWG---------TLEEIEQQQVT-----------LQKRVERDIHQEELT----------------------------KLETELQVLQRRIVDASESSLLIGEEK Escherichia coli B7A
480 TILKQDWG---------SLEEIQQQQAT-----------LQKRNDRDIHQDEVE----------------------------KLEAELQTLQRRIIDAGESSVLIDEENERAH Salmonella enterica erovar Cerro 87
551 SLLKQDWG---------SLEEIKQKHAS-----------LQSKAEFDIHQQEID----------------------------RYNQQLIDIDKQLLKEF Vibrio tasmaniensis 1F-267
551 SLLKQDWG---------SLEEIKQKHAS-----------LQSKAEFDIHQQEID----------------------------RYNRQLIDIDKQLLKEF Vibrionales bacterium C3R12
464 SLLKQDWG---------SYEDIR------------------EKMVNHKHQHSLD----------------------------KNIDKGYDERLGELAAELESLN Bermanella marisrubri RED 65
466 NLLKNDWG---------SKEDIQAAKAAATHTSD-----FKNKLKTELNTESSD----------------------------LHLKSVYDEQLKALTKEYEALS Hahella chejuensis KCTC 2396
460 KVMREDWR---------PMEEIV----------------------ADIEGEE-----------------------------NVENWQYTSESQT Halobellus limi
462 KIFREDWR---------SIEEIT----------------------ADIEGE------------------------------NPERWEYDVKYEEMMQ Halapricum salinum JCM 19729
462 KIFREDWR---------SIEEIT----------------------DDYESE------------------------------DPEQWQYDVEYEEMME Natrinema thermotolerans A29
443 KIFREDWR---------SIEEIT----------------------DEYESE------------------------------DPENWQYDIEYEEMMGQLDR Natronorubrum bangense JCM 10635
479 DVLDKG-----------AFDNLF------------------EAKKFALERERHRRQIELEHIQIKITNSQAESAHSDAVKELQQQQESLEESIAIITKSLKEDGYSSLLIESVENV Alteromonas sp. BL110
473 RCFTTSSRSQGEAIKNAHFKRDLREAVSEGNVAKVKQLTLGDALSINQVENEVKKSS----LDLVNSADNLT------L--ESGIDESNESS--------KAKNWQNMKFQAKDSK Brunnivagina elsteri CCALA 953
501 KTFSR------------HFYDDK-----------------QDALTRAQQMADQKQR---------LSNNRQQ------F--VSVVMEPLACD--------QEGEA Geobacter uraniireducens Rf4
449 EVLKAN-----------AFRTEN------------------EALEFA--RS-----------N-------------------LEDGQEEING----------GEELELIPLTAVEAEED Pseudomonas fluorescens Pfo-1
455 KAIKK------------GYYEDA-----------------DDALRFAKRLQEMRE-----------SDPTQ-----------TTADEEITTD--------APA Streptomyces lividans 1326
480 DVLDKG-----------AFNSMF------------------EAKEFALERERHR-------LHIQLTNDQ-------SL--LPEEQEKIKDKIHMITKCIKEQGYSSLPLETEIVEID Vibrio splendidus ZS-139
```

Supplementary Movies:

Supplementary Movie S1.

Normal mode analysis of *E. coli* B7A DndCDE in complex with DNA.
