# Supplementary figures and images for: Molecular Insight into the Recognition of DNA by the DndCDE Complex in DNA Phosphorothioation
Source: Int J Mol Sci. 2025 Jun 16;26(12):5765. doi: 10.3390/ijms26125765 (PMC12193691; doi:10.3390/ijms26125765)

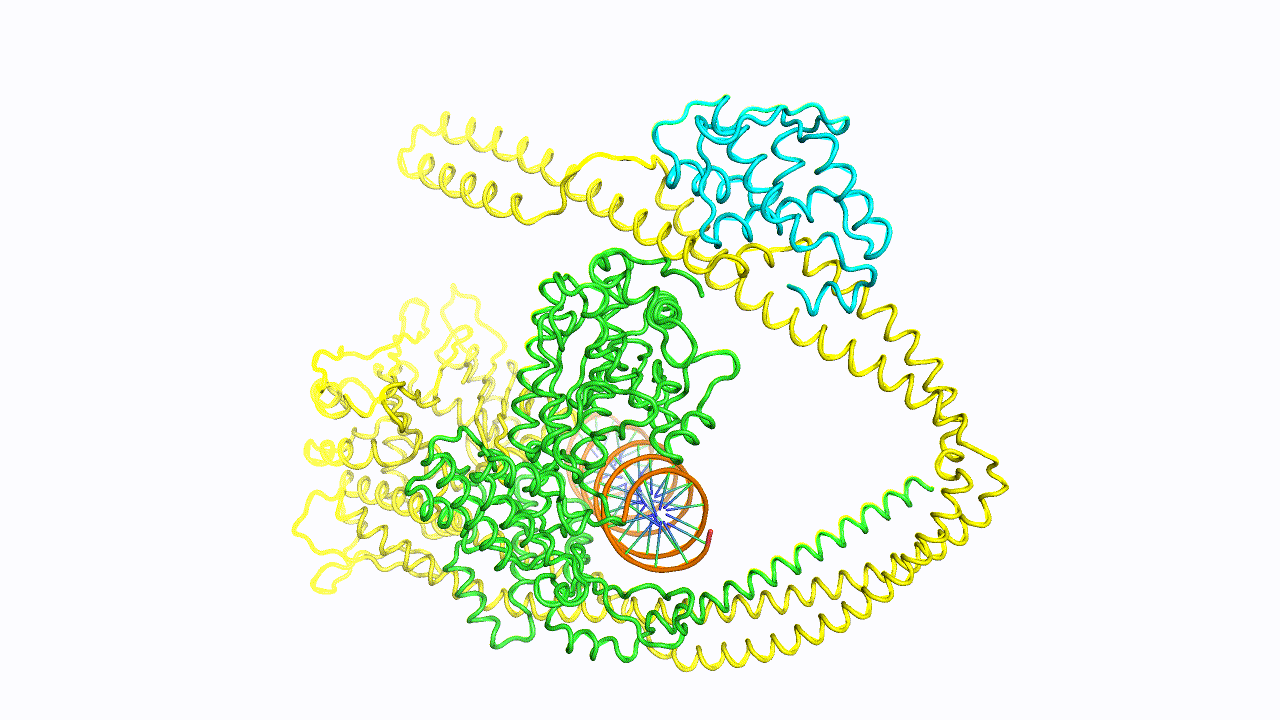

Supplement: Supplementary file 1 [file ijms-26-05765-s001.zip › Supplementary Movie S1.gif]
